# Design and Cohort Characteristics of the Social Spectrum Study: A Multicenter Study of the Autism Spectrum Among Clinically Referred Children

**DOI:** 10.1007/s10803-016-2919-x

**Published:** 2016-10-03

**Authors:** Jorieke Duvekot, Leontine W. ten Hoopen, Geerte Slappendel, Jan van der Ende, Frank C. Verhulst, Ad van der Sijde, Kirstin Greaves-Lord

**Affiliations:** 1 Department of Child and Adolescent Psychiatry/Psychology, Erasmus Medical Center-Sophia Children’s Hospital, Wytemaweg 8, P.O. Box 2060, 3000 CB Rotterdam, The Netherlands; 2 Yulius, Organization for Mental Health, Dordrecht, The Netherlands

**Keywords:** Autism spectrum disorder, Multicenter, Design, Clinical cohort, Attrition, Longitudinal

## Abstract

This paper provides an overview of the design and cohort characteristics of the Social Spectrum Study: a clinical cohort study that used a two-phase sampling design to identify children at risk for ASD. After screening 1281 children aged 2.5–10 years who had been consecutively referred to one of six mental health services in the Netherlands, children who screened positive for ASD (n = 428) and a random selection of screen negatives (n = 240) were invited to participate in diagnostic assessments and questionnaires regarding the child, family and society. A 1-year follow-up was also conducted. Results from this study may contribute to knowledge of the identification and characterization of children with ASD, family processes, and the impact of ASD on the family and society.

## Introduction

Autism Spectrum Disorder (ASD) is a pervasive neurodevelopmental disorder that greatly impacts the functioning of the individual in multiple domains, as well as the family and the broader society (Buescher et al. 2014). The Social Spectrum Study is a prospective clinical cohort study designed to contribute to the understanding of the relationships between ASD characteristics and various child, family and societal factors. In order to enhance generalizability of the findings from this cohort, we systematically screened all children who had been referred to six large mental health services and provided in-depth diagnostic assessments to children who screened positive for ASD as well as to a randomly selected sample who screened negative. This sampling method distinguishes our study from previous studies that usually sampled only children who have an ASD diagnosis or who are considered at risk for ASD. Research has shown that limiting sampling to children with an ASD diagnosis could risk the under-identification of certain subgroups, such as girls (Dworzynski et al. 2012), children with ASD who have normal to high levels of cognitive functioning or subtler symptoms (Kim et al. 2011; Baird et al. 2006), or children of certain ethnic origins (Mandell et al. 2009). Standardized screening and diagnostic methods could help to minimize these biases (Baird et al. 2006).

In line with the current view that ASD represents the extreme end of a continuum of autistic characteristics (Constantino 2011; Lord and Jones 2012; Volkmar and McPartland 2014), we used continuous measures of ASD symptomatology as well as categorical diagnostic assessments of ASD. Research has shown that ASD symptoms are continuously distributed in the general population (Constantino and Todd 2003; Skuse et al. 2009) and that subthreshold ASD symptoms in the general population are related to functional impairment (Skuse et al. 2009). In addition, there is evidence that subclinical levels of ASD symptoms have a similar genetic liability as clinically diagnosed ASD (Colvert et al. 2015). This is also consistent with a general shift in psychiatry from the focus on categorically defined disorders to the dimensional assessment of characteristics that cut across disorders, the Research Domain Criteria (Insel et al. 2010). These findings highlight the importance of examining ASD symptoms in a broader population than only children with a known ASD diagnosis.

The aims of this article are to provide an overview of the aims, design and methods of the Social Spectrum Study, and to present results regarding the characteristics of this cohort as well as factors that influence nonresponse/attrition (i.e., the loss of participants throughout the different phases of the study).

## Aims of the Social Spectrum Study

The Social Spectrum Study investigates how ASD influences and is influenced by various factors on the level of the individual, family, and society. At the individual level, heterogeneity in the core characteristics of ASD as well as co-occurring emotional/behavioral problems greatly complicate diagnosis and treatment of ASD (Constantino and Charman 2016). In order to improve the identification of ASD and the provision of individualized treatments, a better understanding is needed of the performance of screening and diagnostic instruments as well as the relations between ASD and emotional/behavioral difficulties. At the level of the family, the impact of having a child with ASD is evidenced by higher levels of parenting stress and less adequate family functioning in families of children with ASD (Karst and Van Hecke 2012). In addition, parents of children with ASD are at risk for having elevated ASD symptoms and other psychopathology themselves (Sucksmith et al. 2011). Longitudinal research is needed to examine bidirectional influences of child and family factors over time. This could offer insights into how treatment can be tailored to the needs of families in order to improve treatment outcomes. At the societal level, a better understanding of the broader social and economic consequences of having a child with ASD in terms of employment, health care use, and costs is important for the planning of resources (Kogan et al. 2008; Buescher et al. 2014; Leigh and Du 2015).

To address these important issues, the aims of the Social Spectrum Study were:

1. to evaluate the performance of screening and diagnostic instruments for ASD;
2. to investigate the relationships between ASD characteristics and other developmental/mental health problems;
3. to examine the relationships between ASD characteristics of the child and characteristics of the family, such as family functioning, parent–child interaction, parental psychopathology, parenting stress/behavior, and social support;
4. to estimate individual, familial and societal burden of ASD in terms of expenditures on care, lost productivity and quality of life.

**Fig. 1:**
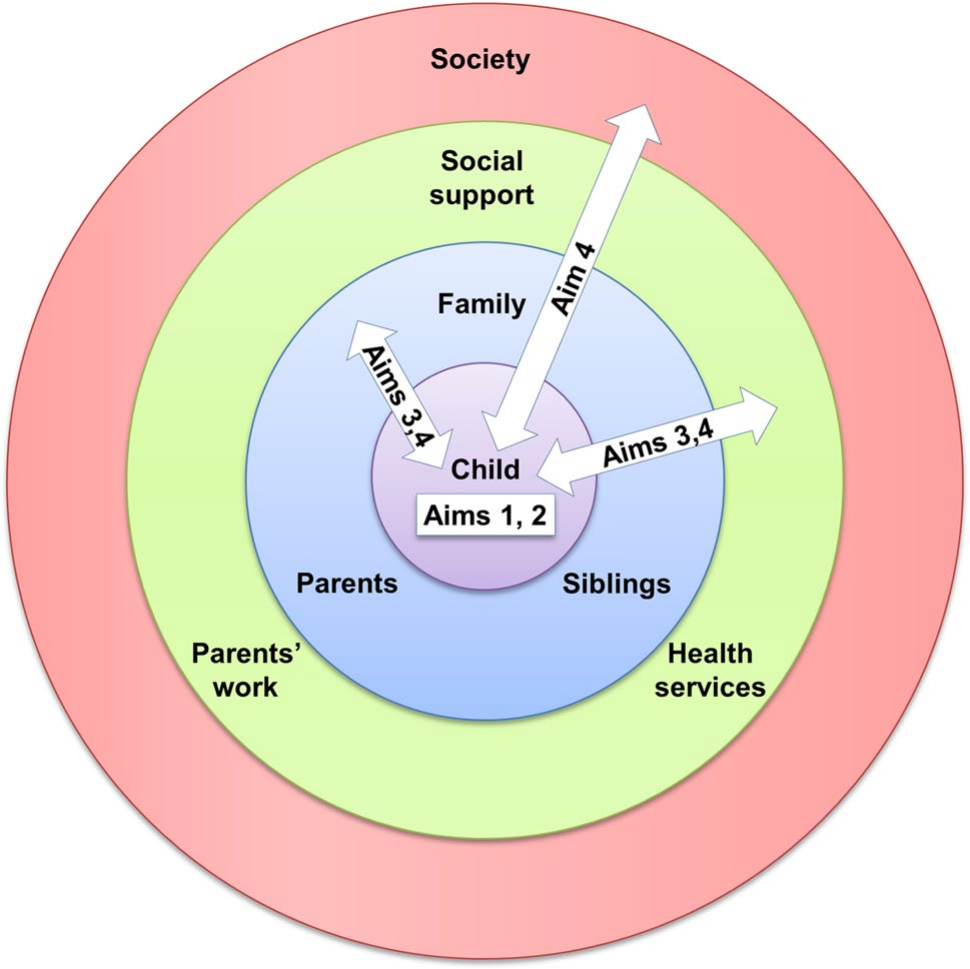
An illustration of how the study’s aims 1 to 4 relate to the different environmental contexts in which the child develops. The figure is based on Bronfenbrenner’s ecological systems theory (Bronfenbrenner 1994)

In line with Bronfenbrenner’s ecological systems theory (Bronfenbrenner 1994), these aims can be linked to the different environmental contexts in which the child is embedded, as depicted in Fig. 1.

## Methods

### Study Design

The present study used a two-phase sampling design (Dunn et al. 1999) to identify children at risk for ASD. In a first phase, all children who had been referred to six large child and adolescent mental health services (CAMHS) in the South-West of the Netherlands were systematically screened for the presence of ASD symptoms using the Social Responsiveness Scale (SRS; Constantino and Gruber 2012) at each site during a period of 6 months falling between April 2011 and July 2012. Children had been referred for a variety of emotional, behavioral and developmental problems. The participating CAMHS were the six largest centers in the South-West of the Netherlands, covering both rural and urban areas. The majority of the CAMHS were secondary services, but also tertiary services participated, including specialized services for children with ASD.

In a second phase, after the completion of the 6-month screening period at a particular site, all children with a positive screen for ASD according to the parent-reported SRS (total raw score ≥75) and a random sample of children with a negative screen for ASD (total raw score <75) were selected for in-depth assessments using select cases in SPSS 20 (IBM Corporation 2011). Of the screen-negative children, we selected approximately 25 % of the screen-negative children aged 4–10 years and to ensure an adequate number of preschoolers—approximately 50 % of the screen-negative children aged 2.5–4 years old. The selection was performed on coded data and selected screen-negative and screen-positive cases were mixed in one file, so the research team did not know whether a selected child had a positive or a negative screen.

The study was approved by the local medical ethics committee (MEC-2011-078) and the participating CAMHS prior to the start of the study. At the time of the in-depth assessments, written informed consent was obtained from the participating parents/caregivers and children aged ≥12 years.

### Measures and Procedures

The measures and procedure at each phase are described in more detail below. Table 1 provides an overview of the measures at different phases of the study.

**Table 1:** Overview of the measures used in the study

Topic	Instrument	Format	Informant/rater	T0	T1	T2
Child characteristics						
ASD symptoms	SRS (Constantino and Gruber 2012)	Questionnaire	Primary caregiver, teacher (only T0)	X	X	X
	3Di-sv (Santosh et al. 2009)	Parent interview	Primary caregiver (informant); clinician/researcher (rater)		X	
	ADOS-2 (Lord et al. 2012)	Child observation	Clinician or researcher		X	
	RBS-R (Bodfish et al. 2000)	Questionnaire	Primary caregiver		X	
	SSP (McIntosh et al. 1999)	Questionnaire	Primary caregiver		X	
Emotional/behavioral problems	CBCL (Achenbach and Rescorla 2000, 2001)	Questionnaire	Primary caregiver	X		X
Cognitive ability	Various IQ tests	Test	Clinician or researcher		X	
Daily living skills	Vineland Screener (van Duijn et al. 2009)	Questionnaire	Primary caregiver		X^a^	X
Emotion regulation	CBQ-SF (Putnam and Rothbart 2006)	Questionnaire	Primary caregiver		X^a^	X^a^
Quality of life	EQ-5D (EuroQol 1990)	Questionnaire	Primary caregiver		X	X
Health care use and expenditures on care	TiC-P (Bouwmans et al. 2012; Hakkaart-van Roijen et al. 2007)	Questionnaire	Primary caregiver	X		X
Health-related absenteeism	TiC-P (Bouwmans et al. 2012; Hakkaart-van Roijen et al. 2007)	Questionnaire	Primary caregiver		X	X
Life events in past year	List of 15 life events	Questionnaire	Primary caregiver			X
Characteristics of primary caregiver						
ASD symptoms	SRS (Constantino and Gruber 2012)	Questionnaire	Self-report & spouse-report		X	
Emotional/behavioral problems	ASR, ABCL (Achenbach and Rescorla 2003)	Questionnaire	Self-report & spouse-report		X	
Social support	VGFO (Veerman et al. 2012)	Questionnaire	Self-report		X	
Marital quality	VGFO (Veerman et al. 2012)	Questionnaire	Self-report		X	
Parenting stress	OBVL (Vermulst et al. 2012)	Questionnaire	Self-report		X	
Personal growth	PGS (Kraaij et al. 2008)	Questionnaire	Self-report		X	
Coping styles	CERQ (Garnefski and Kraaij 2007)	Questionnaire	Self-report		X	
Quality of life (generic)	EQ-5D (EuroQol 1990)	Questionnaire	Self-report		X	
Quality of life (care-related)	CarerQol (Brouwer et al. 2006)	Questionnaire	Self-report		X	
Health care use	TiC-P (Bouwmans et al. 2012; Hakkaart-van Roijen et al. 2007)	Questionnaire	Self-report		X	
Productivity losses	SF-HLQ (van Roijen et al. 1996)	Questionnaire	Self-report		X	
Parenting behavior	PBS-A (Van Leeuwen and Noens 2013)	Questionnaire	Self-report			X
Characteristics of secondary caregiver						
Quality of life	EQ-5D (EuroQol 1990)	Questionnaire	Self-report		X	
ASD symptoms	SRS (Constantino and Gruber 2012)	Questionnaire	Self-report & spouse-report		X	
Emotional/behavioral problems	ABCL (Achenbach and Rescorla 2003)	Questionnaire	Self-report & spouse-report		X	
Characteristics of siblings						
ASD symptoms	SRS (Constantino and Gruber 2012)	Questionnaire	Primary caregiver		X	
Emotional/behavioral problems	CBCL (Achenbach and Rescorla 2000, 2001)	Questionnaire	Primary caregiver		X	
Other family characteristics						
Parent–child interaction (≤5 years)	DPICS (Eyberg et al. 2009)	Observation	Clinician or researcher		X	
Family functioning	FAD (Epstein et al. 1983)	Questionnaire	Primary caregiver		X	X
Family history	FTQ (Mann et al. 1985)	Interview	Primary caregiver (informant)/researcher (rater)		X	

*3Di-sv* short version of the developmental dimensional diagnostic interview, *ADOS-2* autism diagnostic observation schedule, second edition, *ABCL* adult behavior checklist, *ASR* adult self-report, *CarerQol* care-related quality of life, *CBCL* child behavior checklist, *CBQ-SF* short form of the children’s behavior questionnaire, *CERQ* cognitive emotion regulation questionnaire, *DPICS* dyadic parent–child interaction coding system, *FAD* family assessment device, *FTQ* family tree questionnaire, *OBVL* opvoedingsbelastingsvragenlijst [Parenting stress questionnaire], *PBS-A* parent behavior scale for autism spectrum disorders, *PGS* personal growth scale, *RBS-R* repetitive behavior scale-revised, *SF-HLQ* short form of the health and labour questionnaire, *SRS* social responsiveness scale, *SSP* short sensory profile, *TiC-P* trimbos and iMTA questionnaire on costs associated with psychiatric illness, *VGFO* vragenlijst gezinsfunctioneren voor ouders [Questionnaire family functioning for parents]

^a^Only for children aged ≤ 6 years

### T0 Screening

As part of the routine clinical procedure at the participating CAMHS, a screening package containing the ASD screening questionnaire and other questionnaires (see Table 1) was sent to the parents/caregivers prior to the first appointment. In an accompanying letter, parents/caregivers were notified about the study and that they could be invited to participate in further assessments of the study. Although families of all referred children aged 1.5–18 years old received the screening package, we limited further inclusion to children aged 2.5–10 years old to focus only on children of preschool and primary school-age, as these are the ages at which most individuals with ASD are identified.

#### Screening Instrument

The ASD screening instrument used in the present study is the Social Responsiveness Scale (SRS), a 65-item questionnaire that assesses ASD characteristics of children in naturalistic social contexts (Constantino and Gruber 2012). We have chosen the SRS because it is a widely used screening measure for ASD that was specifically developed and validated to assess ASD symptoms across a wide a range of severity in line with the dimensional view of ASD. Therefore, the SRS is also considered useful to identify children with more subtle or less severe forms of ASD, such as Pervasive Developmental Disorder Not Otherwise Specified, in addition to the more classic or severe forms, such as Autistic Disorder (Constantino and Gruber 2012). In contrast, another widely used ASD screening questionnaire, the Social Communication Questionnaire (Berument et al. 1999), was originally developed to provide an indication of the presence of an Autistic Disorder following a categorical definition, rather than to assess variations in symptoms in the broader spectrum. The present study used the school-age version for children aged 4–18 years and the preschool version for children aged 2.5–3 years. The SRS was completed by parents/caregivers as well as by teachers or day care providers. Given the stronger validation base of the parent-reported SRS, we only used the screening result of the parent-reported SRS for selection. A total raw score of 75 or higher on the parent-reported SRS has demonstrated a good sensitivity (0.85) and specificity (0.75) to differentiate between children with ASD and other psychiatric/developmental problems (Constantino and Gruber 2005). Additional support exists for a good reliability and convergent validity of the SRS (Constantino and Gruber 2012; Bölte et al. 2008; Charman et al. 2007; Duvekot et al. 2015). The preschool version is largely similar to the school-age version with a few items adapted to make them more appropriate for preschoolers (Constantino and Gruber 2012).

#### Demographic Information

Information on demographic information of the selected sample was retrieved from patient files. Demographic information of the participants was also collected using online questionnaires. Ethnicity, educational level, and urbanicity were defined according to the Dutch standard classification criteria of Statistics Netherlands (2015). Ethnicity of the child was based on the country of birth of the parents and classified as Dutch, non-Dutch Western, and non-Western. The highest level of completed education of the mother was categorized into three levels: low (primary school or lower vocational education), medium (intermediate vocational education), and high (higher vocational education or university). Because of incomplete data in patient files, maternal educational level was in 20 % of the cases estimated on the basis of mapping maternal occupation to ISCED-97 educational levels (International Labour Organization 2012). Urbanicity was classified as high (≥1500 addresses per square kilometer) or moderate/low (<1500 addresses per square kilometer). Partner status was defined as cohabiting with a partner or not.

### T1 In-Depth Assessments

Selected families for the in-depth assessments received an invitation letter accompanied by an information brochure to inform them about the study and a subsequent phone call after 2 weeks to invite them to participate. Parents could send back a pre-paid reply-card to indicate that they did not want to be contacted further about the study. In case of any questions concerning the study, parents were able to contact the research team and/or an independent psychiatrist. The assessment protocol was identical for the families of children with a positive or negative screen and included well-established standardized diagnostic assessments for ASD and questionnaires assessing several child, family and societal characteristics (see Table 1).

#### Diagnostic Assessment

In line with the gold-standard procedure, a diagnosis of ASD was established based on a standardized parent interview and a standardized observational measure in combination with clinical judgment (Falkmer et al. 2013). Parents were interviewed about the child’s current and past social and communicative behavior and restricted/repetitive behavior using the short version of the Developmental, Dimensional, Diagnostic Interview (3Di-sv; Santosh et al. 2009). In addition, the second edition of the Autism Diagnostic Observation Schedule (ADOS-2; De Bildt et al. 2013; Lord et al. 2012) was used as a standardized, semi-structured observation of the child’s social interaction, communication and restricted/repetitive behavior. Both instruments have good criterion validity (e.g., Santosh et al. 2009; Gotham et al. 2007; Slappendel et al. 2016). The 3Di-sv and ADOS-2 were performed by two different research psychologists who were certified according to the research reliability requirements for administration and coding. They were blind for the SRS scores, the other diagnostic assessment, and any other clinical information. If parents and the child consented, the 3Di-sv was audio-taped and the ADOS-2 was video-taped. The diagnostic assessments were usually scheduled at one of the participating centers near the participant’s home address. If this was not feasible for the parents or child, we offered to administer the 3Di-sv and ADOS-2 during a home visit (12 %) or administered the 3Di-sv by phone (21 %). Additionally, in cases where the 3Di-sv (11 %) or the ADOS-2 (35 %) had already been recently conducted by a trained and certified clinician as part of the clinical evaluation at the CAMHS, the scores on these diagnostic assessments were retrieved from the patient files.

#### Best-Estimate Diagnosis

Following the diagnostic assessments, the two research psychologists who performed the 3Di-sv and the ADOS-2 indicated independently the presence (or absence) of each criterion for ASD according to the DSM-IV-TR and the DSM-5 criteria on a checklist. Subsequently, they discussed their checklists until they reached consensus about the presence of each criterion and a final ASD diagnosis on the basis of information from both the parent interview, the 3Di-sv, and the observation of the child, the ADOS-2. Thus, the consensus diagnosis was based on the information of the 3Di and ADOS, but did not always follow the classification on these instruments, as it formed an integration of information provided by both instruments. Interrater reliability between the indication of an ASD diagnosis based on the DSM-IV-TR symptom checklist that was based on information from each instrument and the consensus diagnosis was good: kappa = 0.81 for the checklist based on the 3Di and kappa = 0.70 for the checklist based on the ADOS. Children received an ASD diagnosis according to the DSM-IV-TR if they met criteria for any pervasive developmental disorder (i.e., autistic disorder, Asperger’s disorder, or pervasive developmental disorder not otherwise specified [PDD-NOS]). In addition, a diagnosis of ASD was made according to the provisional DSM-5 criteria, which were translated into Dutch and back-translated, as our data collection was ongoing during the release of the DSM-5. This procedure for establishing a best-estimate diagnosis was followed in 76 % (*n* = 176) of the cases for which both an ADOS-2 and 3Di-sv was present (*n* = 231). In the other cases one or both diagnostic assessments had been performed by clinicians as part of the clinical evaluation at the CAMHS. In these cases, we used the clinical DSM-IV-TR diagnosis from the patient file established by the clinical staff, including experienced psychologists or psychiatrists, based on the standardized diagnostic assessments in combination with other information assessed during the clinical evaluation.

#### IQ Assessment

IQ scores were obtained from the patient file if the IQ assessment had been conducted within the past 2 years. Frequently used IQ tests were the Wechsler Intelligence Scale for Children, third Dutch edition (WISC-III-NL; Kort 2005), the Wechsler Preschool and Primary Scale of Intelligence, third Dutch edition (WPPSI-III-NL; Hendriksen and Hurks 2009) and the Snijders-Oomen Nonverbal intelligence test (SON-R; Tellegen 1998). If no recent IQ assessment was available, an IQ assessment was conducted by the research team. For children aged 6 years and older, the Wechsler Abbreviated Scale of Intelligence (WASI; Axelrod 2002) was used. For children younger than 6 years old, the WPPSI-III-NL (in verbal children) or the SON-R (in non-verbal children) was administered.

#### Parent–Child Interaction

Parents of children aged 5 years old and younger were asked to participate in a standardized parent–child play observation. The primary caregiver and child were instructed to play together with a set of Duplo toys as they would do at home for 10 min, which was followed by a clean-up task and a gift-delay task. Based on video recordings of the observation, parent–child interaction was coded using a validated coding system, the third edition of the Dyadic Parent–Child Interaction Coding System (DPICS; Eyberg et al. 2009). In addition, emotional self-regulation and co-regulation strategies were coded as reported by Gulsrud et al. (2010).

#### Online Questionnaires

In addition to the diagnostic assessments, the primary caregiver (i.e., the parent/caregiver who spends the most time with the child) received an e-mail with a link to online questionnaires. The online questionnaires for the primary caregiver consisted of three parts: (1) questionnaires about demographic characteristics and child characteristics (i.e., ASD symptoms, daily living skills, emotion regulation, quality of life); (2) questionnaires about characteristics and well-being of the primary caregiver (i.e., mental health, ASD symptoms, social support, marital quality, parenting stress, personal growth, coping, and quality of life) and the broader social/economic impact of their child’s problems (i.e., health care use and costs, productivity losses); (3) questionnaires about family functioning and characteristics of the other parent/caregiver as well as siblings of the child (see Table 1). In addition, a fourth set of questionnaires was sent to the other parent/caregiver (if present) to report on his/her own characteristics and those of the primary caregiver. In order to reduce missing data, parents had to provide an answer to each question in order to continue. The research team was able to track online the progress of completing the questionnaires. If questionnaires were not completed after a few weeks, a researcher contacted the parent/caregiver to ask whether they had any problems filling out the questionnaires and assistance was offered if needed. A hard-copy of the questionnaires was sent if preferred.

### T2 Follow-Up

After approximately a year, the primary caregivers who had participated in at least the first part of the T1 questionnaires (regarding the child’s characteristics) were approached for a follow-up assessment consisting of online questionnaires regarding the child’s characteristics and familial/societal outcomes (e.g., family functioning, parenting behavior and health care use and costs; see Table 1). We approached only primary caregivers who had provided consent to be approached for follow-up research.

### Statistical Analyses

Descriptive statistics (i.e., mean, standard deviation, proportions) for the main demographic and diagnostic variables were computed for the eligible and selected sample and for the T1 and T2 participants. Descriptive statistics for the selected and participating sample were weighted by the inverse of the sampling probability.

Logistic regression analyses were used to examine predictors of attrition at T1. Participation was predicted by age and gender of the child, clinical characteristics (i.e., SRS parent and teacher total raw score, CBCL total problems score, full scale IQ, referral to secondary versus tertiary services, referral reason, and ASD diagnosis of the child before referral) and demographic characteristics (i.e., ethnicity of the child, maternal and paternal age, partner status, maternal educational level, and urbanicity). The SRS, CBCL and full scale IQ scores were transformed to z-scores. Missing data in the predictor variables ranged from 0 to 35 % (10 out of 15 variables had ≤10 % missing data). Since IQ assessments were more likely to be performed as part of the clinical procedure in children who were suspected of having cognitive problems, whereas researchers performed IQ assessments in the participating children regardless of cognitive problems, we only used IQ scores derived from patient files in the analyses. To examine loss to follow-up from T1 to T2, a similar logistic regression analysis was performed predicting participation at T2 among the caregivers who completed the questionnaires at T1. In these analyses, all predictor variables had less than 10 % missing data. In order to account for missing data in all attrition analyses, we used multiple imputations with 30 imputed datasets using SPSS version 20.0 (IBM, Armonk, NY).

Finally, we examined frequencies and descriptive statistics of children who were diagnosed with ASD according to the DSM-IV-TR criteria. In addition, we examined the convergence between the DSM-IV and DSM-5 ASD diagnoses. We used multivariate analysis of variance (MANOVA) with post-hoc Games-Howell tests (because of unequal group variances) to compare core ASD symptom levels on the 3Di and ADOS of children who were diagnosed with ASD according to the DSM-IV, but not according to the DSM-5 (labelled ASD-divergent) with those of children who met criteria for ASD according to both the DSM-IV and DSM-5 (labelled ASD-convergent) and children who were classified as non-ASD according to both the DSM-IV and DSM (labelled non-ASD). There were no children who met DSM-5 criteria for ASD but not DSM-IV criteria, so this group was not included in the analysis.

## Results

### Sample Inclusion

**Fig. 2:**
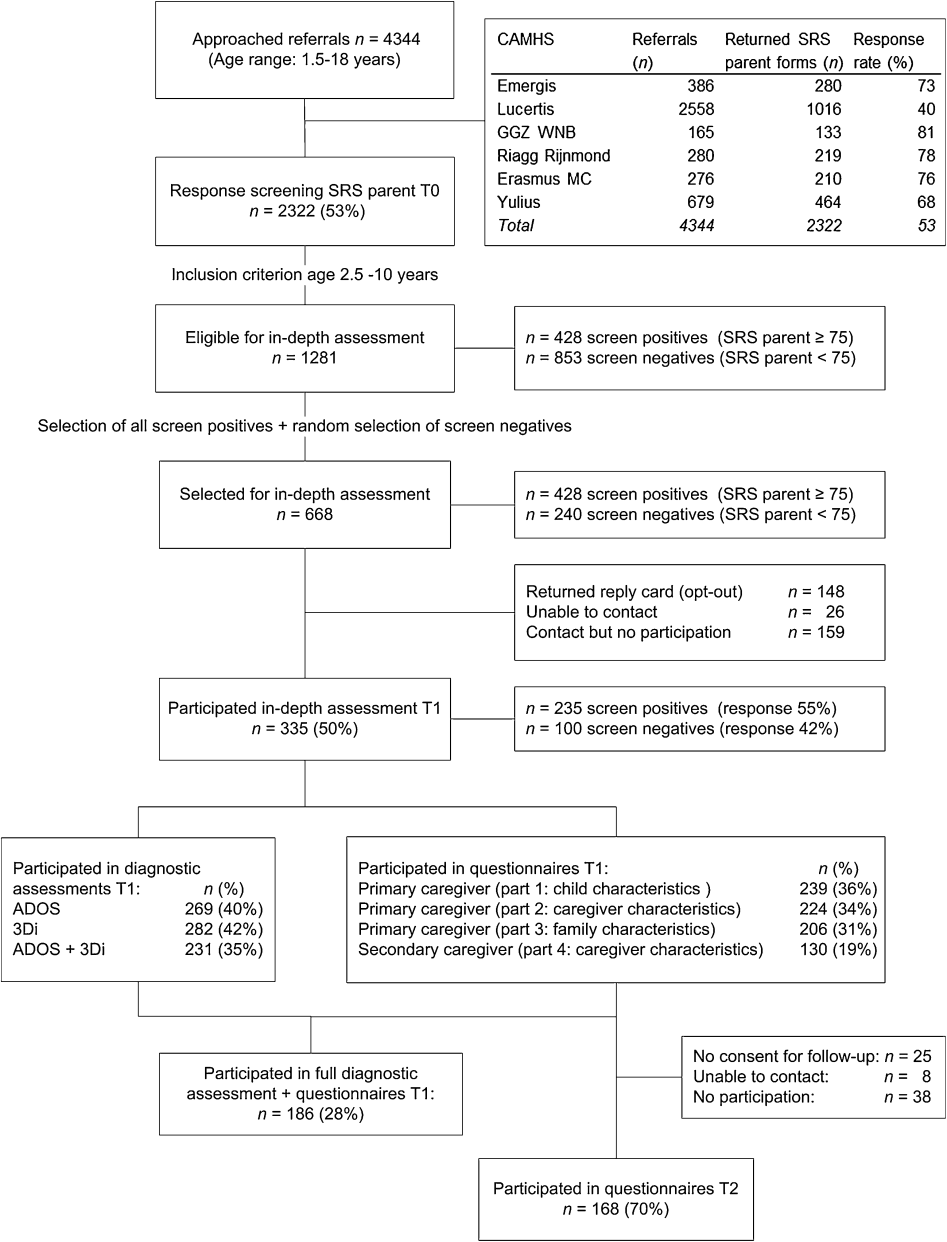
Flow of the participants through different phases of the study

The flow of participants through different phases of the study is shown in Fig. 2. Since it was not possible to retrieve the exact number of children in the particular age range of 2.5–10 years old who had been referred to the CAMHS during the screening phase, we estimated the response rate of the parent-reported SRS by dividing the total number of returned parent-reported SRS questionnaires for all children aged 1.5–18 years old by the total number of referrals during the 6-month screening phase at each CAMHS. This resulted in a response rate of 68–81 % for the parent-reported SRS among the participating CAMHS, except for one CAMHS with a response rate of 40 % (see Fig. 2). Because we lacked a reliable overall registry of referrals that received the screening questionnaire at this particular CAMHS, we had to estimate this response rate based on several separate registries which possibly included sites that did not sent the screening package. Therefore, this response rate should be considered with caution, probably being a conservative estimate.

In the screening phase, we received 1281 completed parent reports of the SRS for children aged 2.5–10 years (*M* age = 6.9, *SD* = 2.2). Of these children, 428 (33 %) screened positive (total raw score ≥75) on the parent report SRS and 853 (67 %) screened negative. The proportion of children with a positive screen was similar for children screened with the preschool version (35 %) versus the school-age version of the SRS (33 %), *χ*²(1) = 0.18, *p* = .67. The mean age of the children who screened positive did not differ significantly from that of the children with a negative screen (*t*(799.21) = − 0.62, *p* = .54). A slightly higher proportion of boys had a positive screen (35 %) compared with girls (30 %), but this was not significant (*χ*²(1) = 3.36, *p* = .07). Parent reports were completed in 88 % of the cases by the biological mother, in 9 % by the biological father, and in 7 % by another caregiver (adoptive/stepparent). For 1089 (85 %) of the children for whom a parent completed the SRS, a teacher (91 %) or a day care provider/counselor (9 %) also completed the SRS.

All 428 children who scored 75 or higher on the parent-reported SRS and a random selection of 240 children who scored below this cut-off were selected for in-depth assessments. This random selection consisted of 203 out of the 789 (26 %) school-age children who screened negative and 37 out of the 64 (58 %) preschoolers who screened negative. Of the 668 selected children, 148 (22 %) families sent back a reply card indicating that they did not want to be contacted about the study and we were unable to reach an additional 26 (4 %) families. Families of 335 children participated in at least one assessment (i.e., ADOS-2, 3Di-sv or online questionnaires) at T1 (response rate 50 %). For 320 children we had at least one diagnostic assessment (ADOS-2 or 3Di-sv; response rate 48 %), for the remaining 15 cases only online questionnaires were available. Full diagnostic assessment was available for 231 children (ADOS-2 and 3Di-sv; response rate 35 %). Participation rates for the different parts of the online questionnaires at T1 are shown in Fig. 2. For 188 cases (28 %), we had full diagnostic assessments as well as questionnaire data regarding child characteristics by the primary caregiver (i.e., the first set of questionnaires). Children were on average 7.5 years old (*SD* = 2.4, range 2–12) at the time of the T1 diagnostic assessments and 7.9 years old (*SD* = 2.4, range 3–12) at the time of the T1 online questionnaires.

Of the 239 primary caregivers who completed at least the first part of the T1 questionnaires, 214 (90 %) provided consent to be contacted for follow-up assessments (T2). At T2, 168 primary caregivers (70 %) completed the online questionnaires. The average age of the children at the time of the T2 assessment was 8.8 years old (*SD* = 2.3, range 4–13).

Characteristics of the screened sample at T0 and of the participants at T1 and T2 are presented in Table 2.

**Table 2:** Sample characteristics

	Eligible T0 (n = 1,281)		Selected T0 (n = 668)		Participants T1 diagnostic assessments (n = 320)		Participants T1 questionnaires (n = 239)		Participants T2 questionnaires (n = 168)	
	M	SD	M	SD	M	SD	M	SD	M	SD
Gender, male (%)	69.1	–	69.9	–	72.0	–	68.9	–	67.8	–
Age screening (years)	6.9	2.2	6.9	2.3	6.8	2.3	7.0	2.3	7.0	2.2
SRS										
Parent report total	63.6	28.7	63.0	29.7	68.8	28.7	68.5	27.9	69.7	27.6
Teacher report total	63.5	30.2	62.3	31.2	66.1	30.5	64.9	30.2	66.0	30.1
CBCL										
Internalizing	61.5	10.7	61.5	9.9	63.4	9.5	63.7	9.4	63.3	9.8
Externalizing	62.4	11.4	62.4	11.0	64.0	10.4	63.9	10.7	64.2	11.0
Full scale IQ^a^	–	–	94.0	17.3	94.9	17.5	92.9	17.7	96.3	18.4
Tertiary CAMHS (%)	15.5	–	15.5	–	24.3	–	17.0	–	20.0	–
ASD before referral (%)	–	–	8.0	–	7.8	–	7.3	–	8.4	–
Referral reason ASD (%)	–	–	23.1	–	29.5	–	24.1	–	28.0	–
Child ethnicity (%)										
Dutch	–	–	78.6	–	77.7	–	81.2	–	89.1	–
Non-Dutch Western	–	–	3.8	–	4.8	–	6.0	–	3.2	–
Non-Western	–	–	17.6	–	17.5	–	12.7	–	7.7	–
Maternal age	–	–	36.8	5.5	36.5	5.4	36.8	5.3	37.3	5.0
Paternal age	–	–	39.6	5.8	39.2	5.8	39.4	5.7	39.8	5.5
Maternal education (%)										
Low	–	–	26.4	–	27.9	–	27.1	–	26.7	–
Medium	–	–	51.3	–	50.5	–	48.6	–	47.7	–
High	–	–	22.3	–	21.6	–	24.3	–	25.6	–
Married/cohabiting (%)	–	–	75.6	–	77.8	–	77.9	–	87.1	–
High urbanicity (%)	–	–	69.0	–	67.0	–	63.6	–	62.8	–

Reported frequencies are unweighted; other descriptive statistics (*M* and percentages) are weighted by the inverse of the sampling probability

*SRS* social responsiveness scale, *CBCL* child behavioral checklist, *CAMHS* child and adolescent mental health service

^a^Only IQ scores from patient files are reported

### Attrition Analyses

Since results differed for participation in the diagnostic assessments (defined as 3Di or ADOS) versus participation in the questionnaires (defined as completion of at least the first part of the online questionnaires by the primary caregiver) at T1, we present the results of these attrition analyses separately in Table 3. After accounting for other clinical and demographic characteristics, the only significant predictor of participation in diagnostic assessments at T1 was a referral to a specialized tertiary mental health service. In addition, primary caregivers were more likely to participate in the questionnaires at T1 if the child showed higher levels of internalizing problems. Participation in the online questionnaires at T2 by the primary caregivers who completed the online questionnaires at T1 was mostly determined by demographic characteristics. Caregivers who did not cohabit with a partner and caregivers who had a child of a non-Dutch ethnicity were more likely to be lost to follow-up.

**Table 3:** Logistic regression models predicting participation at T1 (diagnostic assessments and questionnaires) and T2 (questionnaires)

	Diagnostic assessments T1 (*n* = 320)		Questionnaires T1 (*n* = 239)		Questionnaires T2 (*n* = 168)	
	OR	95 % CI	OR	95 % CI	OR	95 % CI
Child’s gender (boys vs. girls)	1.28	[0.88, 1.87]	1.01	[0.68, 1.49]	0.89	[0.41, 1.90]
Child’s age (years)	0.99	[0.92, 1.08]	0.98	[0.91, 1.07]	0.86	[0.73, 1.03]
SRS parent total score	1.21	[0.95, 1.55]	1.17	[0.91, 1.49]	0.98	[0.60, 1.60]
SRS teacher total score	1.07	[0.89, 1.30]	1.07	[0.88, 1.30]	0.95	[0.64, 1.39]
CBCL internalizing	1.21	[0.96, 1.53]	1.29*	[1.01, 1.64]	0.89	[0.56, 1.40]
CBCL externalizing	1.02	[0.82, 1.27]	0.96	[0.77, 1.20]	1.23	[0.80, 1.90]
Full scale IQ^a^	1.05	[0.86, 1.28]	1.2	[0.98, 1.47]	1.04	[0.73, 1.47]
CAMHS (tertiary vs. secondary)	2.53***	[1.60, 3.99]	0.94	[0.60, 1.47]	0.59	[0.24, 1.41]
ASD diagnosis before referral	0.64	[0.36, 1.11]	0.98	[0.56, 1.71]	1.41	[0.46, 4.31]
Referral reason (ASD vs. other)	0.71	[1.06, 1.06]	1.08	[0.72, 1.62]	0.48	[0.21, 1.11]
Child’s ethnicity		–		–		–
Dutch	REF	–	REF	–	REF	–
Western non-Dutch	1.25	[0.59, 2.65]	1.96	[0.92, 1.96]	0.30*	[0.09, 0.98]
Non-Western	0.93	[0.57, 1.51]	0.74	[0.45, 0.74]	0.36*	[0.14, 0.94]
Maternal age	1	[0.96, 1.05]	1.01	[0.97, 1.01]	1.03	[0.94, 1.13]
Paternal age	0.99	[0.95, 1.03]	1	[0.96, 1.00]	1.06	[0.98, 1.14]
Partner vs. no partner	1.14	[0.75, 1.72]	1.25	[0.81, 1.25]	4.27***	[1.93, 9.41]
Maternal education		–		–		–
Low	1.08	[0.64, 1.84]	1.11	[0.65-1.11]	0.44	[0.17, 1.18]
Medium	1.03	[0.64, 1.65]	0.86	[0.54, 0.86]	0.66	[0.27, 1.59]
High	REF	–	REF	–	REF	–
Urbanicity (high vs. low)	0.95	[0.63, 1.42]	1.13	[0.76, 1.13]	0.96	[0.47, 1.96]
*Nagelkerke R* ^*2*^ * of model*	*0.12*	*[0.10, 0.15]*	*0.07*	*[0.05, 0.09]*	0.24	[0.17, 0.31]

Non-participants are used as reference

*REF* reference group, *SRS* social responsiveness scale, *CBCL* child behavioral checklist, *CAMHS* child and adolescent mental health service

*p < .05, **p < .01, ***p < .001

^a^Only Full Scale IQ scores from the patient file were used in the analyses

### ASD Ascertainment

#### DSM-IV-TR

Within the sample of children for whom full diagnostic assessment was available (3Di-sv and ADOS-2, *n* = 231), 130 (56 %) were assigned a best-estimate consensus diagnosis of ASD according to the DSM-IV-TR criteria (autistic disorder, *n* = 72; Asperger’s disorder, *n* = 8; PDD-NOS, *n* = 50). Of the 130 children with a best-estimate diagnosis of ASD according to the DSM-IV-TR, 69 % met criteria for an autism/ASD classification on the ADOS-2, 69 % met criteria for ASD on the 3Di-sv, and 47 % met ASD criteria on both instruments. For the 101 non-ASD children, these proportions were 23 % for the ADOS-2, 19 % for the 3Di-sv, and 5 % for both. Children who did not receive an ASD diagnosis had a range of psychiatric diagnoses as reported in the patient file, with ADHD being the most common diagnosis (39 %), followed by anxiety/mood disorders (11 %). Of the children with ASD, 89 (69 %) scored in the clinical range on at least one of the DSM-oriented subscales of the CBCL, indicating the presence of psychiatric comorbidity. Several child and family characteristics of the ASD and non-ASD sample are presented in Table 4.

**Table 4:** Characteristics of the ASD and non-ASD sample

	ASD			Non-ASD		
	N	M (SD)/n(%)	Range	N	M (SD)/n(%)	Range
Child characteristics						
Gender (% boys)	130	106 (81.5 %)	–	101	61 (60.4 %)	–
Age at T1 (years)	130	7.6 (2.3)	2–12	101	7.7 (2.5)	3–12
Ethnicity (% Dutch)	128	104 (81.3 %)	–	101	74 (73.3 %)	–
Full scale IQ	123	96.4 (17.6)	50–141	94	96.1 (17.2)	50–130
Intellectual disability^a^	127	17 (13.4 %)	–	100	9 (9 %)	–
SRS parent total	130	93.3 (26.0)	26–152	101	74.8 (28.3)	16–136
SRS teacher total	114	75.6 (30.6)	4–153	90	62.8 (26.1)	12–121
CBCL internalizing problems	117	67.1 (9.8)	34–88	99	66.0 (9.4)	34–87
CBCL externalizing problems	117	67.1 (10.6)	40–97	99	68.1 (10.3)	44–92
CBCL clinical cut-offs on DSM-scales						
Affective problems		53 (45.3 %)	–		42 (42.4 %)	–
Anxiety problems		40 (34.2 %)	–		30 (30.3 %)	–
Somatic problems^b^		11 (13.6 %)	–		9 (13.4 %)	–
ADHD problems		49 (41.9 %)	–		43 (43.4 %)	–
Oppositional defiant problems		48 (41.0 %)	–		50 (50.5 %)	–
Conduct problems^b^		27 (33.3 %)	–		31 (45.6 %)	–
ADOS social affect CSS	130	5.3 (2.5)	1–10	101	2.5 (1.9)	1–8
ADOS restricted/repetitive CSS	130	4.4 (2.8)	1–10	101	2.5 (2.2)	1–10
ADOS total CSS	130	6.1 (2.4)	1–10	101	3.2 (2.3)	1–10
3Di reciprocal social interaction	130	13.0 (5.0)	2–26	101	6.8 (5.0)	0–20
3Di communication	130	12.5 (4.4)	1–23	101	8.0 (4.7)	0–20
3Di repetitive/stereotyped	130	3.1 (2.3)	0–11	101	1.4 (1.6)	0–8
Family characteristics						
Maternal education (% high)	122	29 (23.8 %)	–	96	21 (21.9 %)	–
Two-parent household, %	128	108 (84.4 %)	–	100	76 (76.0 %)	–
Urbanicity (% high)	124	87 (70.2 %)	–	101	66 (66.7 %)	–
Parenting stress (OBVL)	97	61.5 (15.2)	34–105	79	59.9 (15.2)	35–100
Family functioning (FAD)	92	21.3 (4.8)	12–34	74	21.6 (5.6)	12–35

Diagnosis of ASD was based on the DSM-IV-TR criteria

*3Di* developmental, dimensional and diagnostic interview, *ADOS* autism diagnostic observation schedule, *CBCL* child behavioral checklist, *CSS* calibrated severity scores, *FAD* family assessment device, *OBVL* opvoedingsbelastingsvragenlijst [Parenting stress questionnaire], *SRS* social responsiveness scale, *VGFO* vragenlijst gezinsfunctioneren voor ouders [Questionnaire family functioning for parents]

^a^Intellectual disability was defined as an Verbal IQ, Nonverbal IQ or Full scale IQ < 70 or a DSM-IV-TR axis classification of intellectual disability (code 317, 318, 319)

^b^Only present in the CBCL/6–18 version

#### DSM-5

For a subsample of 176 children for whom the research psychologists performed both diagnostic assessments, we also formed a best-estimate consensus diagnosis of ASD according to the DSM-5 criteria: 65 (37 %) were diagnosed with ASD according to the DSM-5. In 81 % of the cases (65 ASD and 78 non-ASD), the DSM-IV-TR and DSM-5 diagnosis agreed (Kappa = 0.64). However, for 33 children (19 %) the DSM-IV-TR and DSM-5 disagreed: these children met ASD criteria according to the DSM-IV-TR but not according to the DSM-5. Of the children with a DSM-IV diagnosis of autistic disorder, 92 % (56 out of 61) also had a diagnosis of ASD according to the DSM-5. In addition, 4 of the 5 (80 %) children with a DSM-IV diagnosis of Asperger’s syndrome had a DSM-5 ASD diagnosis. In contrast, of the children with a DSM-IV diagnosis of PDD-NOS, only 16 % (5 out of 32) met criteria for a DSM-5 ASD diagnosis.

**Fig. 3:**
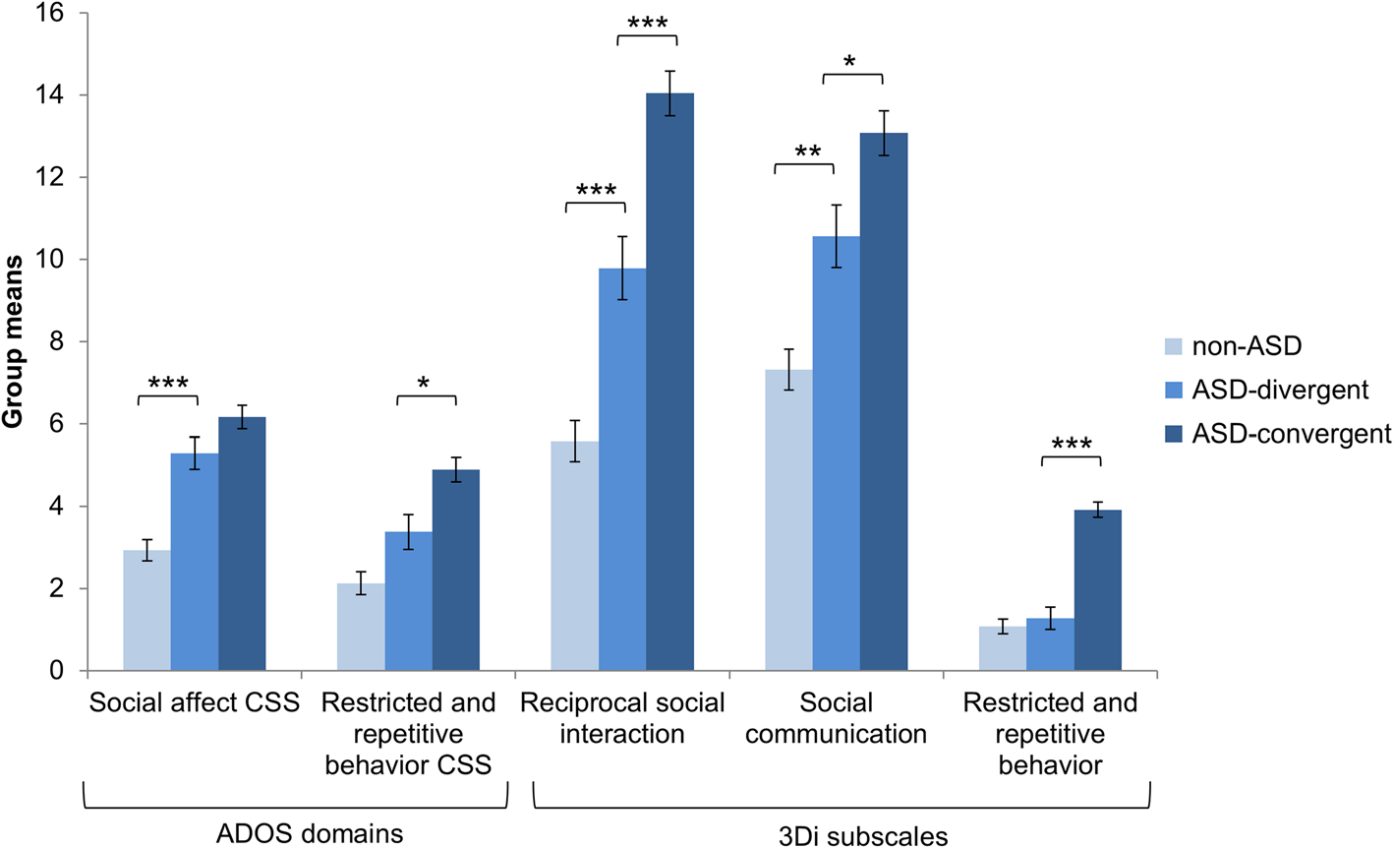
Mean scores on the Autism Diagnostic Observation Schedule (ADOS) and the Developmental, Dimensional and Diagnostic Interview (3Di) in children who met DSM-IV but not DSM-5 criteria for ASD (ASD-divergent) vs. children who met both DSM-IV and DSM-5 criteria (ASD-convergent) and children who were classified as non-ASD according to both DSM-IV and DSM-5 (non-ASD). *CSS* calibrated severity score. *Error bars* represent standard errors. *Asterisk* indicate significant group differences. *p < .05, **p < .01, ***p < .001

As would be expected, there were significant differences in ADOS and 3Di scores between children who met DSM-IV but not DSM-5 criteria for ASD (ASD-divergent), children who met both DSM-IV and DSM-5 criteria (ASD-convergent), and children who were classified as non-ASD according to both DSM-IV and DSM-5 (non-ASD), *F*(10, 340) = 19.76, *p* < .001. As shown in Fig. 3, the ASD-divergent had significantly lower levels of restricted and repetitive behaviors (RRB) on the ADOS and 3Di than the ASD convergent group. The RRB scores of the ASD-divergent group were similar to those of the non-ASD group. On the ADOS, the social impairment scores of the ASD-divergent group were not different from those of the ASD-convergent group; both groups had higher scores than the non-ASD group. On the 3Di, the highest levels of social interaction and communication impairments were found in the ASD-convergent group, followed by the ASD-divergent group, and then the non-ASD group.

## Discussion

The Social Spectrum Study is prospective cohort of clinically referred children enriched for children with ASD that provides the opportunity to examine a wide range of child, family, and societal factors in relation to ASD symptomatology. This paper described the aims and methods of this study and provided some details regarding attrition and characteristics of the participating children and their families.

### Attrition

Whereas participation in the first assessments was mainly determined by clinical characteristics (i.e., referral to a tertiary service, higher levels of internalizing problems), participation in the questionnaires by primary caregivers at the 1-year follow-up was mainly determined by demographic characteristics (i.e., not having a partner, non-Dutch ethnicity of child). A likely explanation for the finding that children who had been referred to tertiary mental health services were more likely to participate in the diagnostic assessments, is that these assessments were more often performed as part of the clinical evaluation in tertiary than in secondary CAMHS. In addition, caregivers of children with higher levels of internalizing problems may have been more likely to complete the questionnaires at T1 because they could better relate to the relevance of the study than caregivers of children with less problems. Moreover, internalizing problems might place less burden on caregivers than other types of psychopathological problems (Davis and Carter 2008) and therefore interfere less with participation. Attrition at the 1-year follow-up (T2) of caregivers who did not cohabit with a partner could reflect that these caregivers experienced greater difficulty in finding the time to complete the questionnaires in the previous assessment. In addition, caregivers of children from other ethnicities may have been less likely to participate in the follow-up because they experienced more difficulties in completing the questionnaires due to problems with the language or topics discussed. In contrast to several general population studies (Stoltenberg et al. 2010; Jaddoe et al. 2012), we did not find that lower maternal education increased the risk of attrition during any phase of the study.

### Characteristics of the Sample

Our sample is relatively high-functioning in terms of intellectual ability. Only 13 % of the ASD sample had an intellectual disability compared to an estimate of 32–55 % in recent epidemiological studies (Baird et al. 2006; Baio 2012). Consistent with the literature (Simonoff et al. 2008), we found high rates of clinically elevated co-occurring psychiatric problems based on a parent-reported questionnaire, ranging between 33 and 45 % for affective problems, anxiety problems, ADHD problems, oppositional defiant problems and conduct problems. At first sight, children with ASD seemed to have similar levels of parenting stress and family functioning as the non-ASD group. This could be explained by the fact that ASD is a very heterogeneous group showing a large variation in ASD symptom severity, intellectual functioning and co-occurring emotional and behavioral problems; characteristics that are shared with the comparison group (Hayes and Watson 2013). Moreover, some studies suggested that parenting stress and family functioning in families of children with ASD are particularly related to co-occurring emotional and behavioral problems (e.g., Herring et al. 2006; Lecavalier et al. 2006; Davis and Carter 2008). That is why it is important that we also assessed variation in ASD symptoms and emotional/behavioral problems on a dimensional scale. In addition, parent characteristics and resources, such as being a single parent, social support, and coping strategies need to be accounted for as well (Zaidman-Zait et al. 2016; Karst and Van Hecke 2012). In future papers, we will more thoroughly investigate these complex interrelations between child, parent, and family characteristics. This could help to identify families who need interventions to promote more optimal family functioning, which in turn may lead a more optimal child development. We cannot yet provide information about the societal factors we assessed (e.g., health care costs, productivity losses), as this data is still being processed.

Although this was not a specific aim of this study, in light of the discussion around the sensitivity of DSM-5 criteria for ASD (e.g., Tsai 2012), it is interesting to note that in our study a group of children with ASD according to the DSM-IV-TR criteria did not meet DSM-5 criteria for ASD. This particularly concerned children with a DSM-IV PDD-NOS diagnosis, of which only 16 % also had a DSM-5 ASD diagnosis. In contrast, almost all (92 %) children with a DSM-IV diagnosis of autistic disorder had a DSM-5 ASD diagnosis. Consistently, Smith et al. (2015) reported in a systematic review that in half of the studies less than 25 % of the children with PDD-NOS met DSM-5 criteria for ASD, whereas rates were much higher for children with an autistic disorder. Compared to children with an ASD diagnosis according to both the DSM-IV and DSM-5, children with a DSM-IV ASD diagnosis who did not meet DSM-5 ASD criteria were characterized by relatively low levels of RRB symptoms and milder levels of social communication impairment in our study. As they still showed significant impairments in the social domain on the ADOS and 3Di compared to the non-ASD group, these children might be eligible for a diagnosis of a Social Communication Disorder (SCD). This new and controversial diagnostic category describes social communication impairments similar to those of ASD without the RRB symptoms (American Psychiatric Association 2013). Although we did not evaluate children using the SCD criteria in our study, a previous study found that many children who did not maintain an ASD diagnosis using DSM-5 criteria met criteria for SCD (Kim et al. 2014).

### Strengths and Limitations

A particular strength of this study is that we systematically screened all children referred to one of six mental health services for ASD and subsequently performed standardized diagnostic assessment in both screen-positive and screen-negative children. Using this ascertainment method, we aimed to overcome certain biases that may be present when only recruiting children with an established diagnosis. Besides the delineation of a well-characterized ASD sample using categorical diagnostic instruments, we also captured a wide range of ASD symptom severity in the total cohort of clinically referred children using continuous measures. Another strength is that we used various measures and informants to assess a wide range of characteristics regarding the child, family and society, allowing the investigation of a broad scope of topics. Lastly, we conducted a follow-up assessment that enables the investigation of longitudinal relations.

Findings from this study should also be interpreted in the light of some limitations. In addition to evidence of selective attrition, possible biases, which we could not investigate, may already have been present in the referral process. Thus, findings from this cohort cannot be generalized to children at risk for ASD who are not referred to mental health services (i.e., the general population). In addition, participation in full assessments was rather low (28 %), limiting the number of children with a consensus diagnosis of ASD for whom we have in-depth information on a large variety of child, family, and societal factors. However, as we stated earlier, it is also of interest to investigate these factors in our larger cohort, including children with subclinical levels of ASD symptomatology. Finally, because some of the diagnostic assessments were integrated in the clinical procedure, we could not follow the same procedure for establishing a best-estimate diagnosis for all participants in the study.

## Conclusion

In conclusion, we obtained a cohort of clinically referred children that includes a well-characterized sample of children with ASD but also allows a dimensional approach of examining relationships in a broader group of clinically referred children with varying levels of ASD symptoms. Given the wide range of child, family and societal factors assessed, this study has the potential to contribute to the understanding of (1) the performance of screening and diagnostic instruments for ASD; (2) the relations between ASD symptomatology and other developmental/mental health problems; (3) the characteristics of families of children with ASD symptomatology; (4) the societal impact of ASD symptomatology. We invite all researchers interested in collaboration to contact Kirstin Greaves-Lord (k.greaves-lord@erasmusmc.nl).

## References

[CR1] Achenbach T. M., Rescorla L. A. (2000). Manual for the ASEBA preschool forms & profiles.

[CR2] Achenbach T. M., Rescorla L. A. (2001). Manual for the ASEBA school-age forms & profiles.

[CR3] Achenbach T. M., Rescorla L. A. (2003). Manual for the ASEBA adult forms & profiles.

[CR4] American Psychiatric Association (2013). Diagnostic and statistical manual of mental disorders 5 (DSM-5).

[CR5] Axelrod B. N. (2002). Validity of the Wechsler abbreviated scale of intelligence and other very short forms of estimating intellectual functioning. Assessment.

[CR6] Baio, J. (2012). Prevalence of autism spectrum disorders: Autism and developmental disabilities monitoring network, 14 Sites, United States, 2008. Morbidity and mortality weekly report. Surveillance summaries. Volume 61, Number 3. *Centers for Disease Control and Prevention*. 22456193

[CR7] Baird G., Simonoff E., Pickles A., Chandler S., Loucas T., Meldrum D. (2006). Prevalence of disorders of the autism spectrum in a population cohort of children in South Thames: The Special needs and autism project (SNAP). Lancet.

[CR8] Berument S. K., Rutter M., Lord C., Pickles A., Bailey A. (1999). Autism screening questionnaire: Diagnostic validity. British Journal of Psychiatry.

[CR9] Bodfish J. W., Symons F. J., Parker D. E., Lewis M. H. (2000). Varieties of repetitive behavior in autism: comparisons to mental retardation. Journal of Autism and Developmental Disorders.

[CR10] Bölte S., Poustka F., Constantino J. N. (2008). Assessing autistic traits: cross-cultural validation of the social responsiveness scale (SRS). Autism Research.

[CR11] Bouwmans C., Schawo S., van HakkaartRoijen L. (2012). Handleiding Vragenlijst TiC-P voor kinderen [manual TiC-P questionnaire for children].

[CR12] Bronfenbrenner U. (1994). Ecological models of human development. International Encyclopedia of Education.

[CR13] Brouwer W. B., van Exel N. J., van Gorp B., Redekop W. K. (2006). The CarerQol instrument: A new instrument to measure care-related quality of life of informal caregivers for use in economic evaluations. Quality of Life Research.

[CR14] Buescher A. V., Cidav Z., Knapp M., Mandell D. S. (2014). Costs of autism spectrum disorders in the United Kingdom and the United States. JAMA Pediatrics.

[CR15] Charman T., Baird G., Simonoff E., Loucas T., Chandler S., Meldrum D. (2007). Efficacy of three screening instruments in the identification of autistic-spectrum disorders. British Journal of Psychiatry.

[CR16] Colvert E., Tick B., McEwen F., Stewart C., Curran S. R., Woodhouse E. (2015). Heritability of autism spectrum disorder in a UK population-based twin sample. JAMA Psychiatry.

[CR17] Constantino J. N. (2011). The quantitative nature of autistic social impairment. Pediatric Research.

[CR18] Constantino J. N., Charman T. (2016). Diagnosis of autism spectrum disorder: Reconciling the syndrome, its diverse origins, and variation in expression. Lancet Neurology.

[CR19] Constantino J. N., Gruber C. P. (2005). Social Responsiveness Scale (SRS).

[CR20] Constantino J. N., Gruber C. P. (2012). Social Responsiveness Scale, Second Edition (SRS-2).

[CR21] Constantino J. N., Todd R. D. (2003). Autistic traits in the general population: A twin study. Archives of General Psychiatry.

[CR22] Davis N. O., Carter A. S. (2008). Parenting stress in mothers and fathers of toddlers with autism spectrum disorders: Associations with child characteristics. Journal of Autism and Developmental Disorders.

[CR23] De Bildt A., Greaves-Lord K., De Jonge M. (2013). ADOS-2: Autisme diagnostisch observatieschema. Handleiding. [ADOS 2nd edition: Autism diagnostic observation schedule. Dutch manual.].

[CR24] Dunn G., Pickles A., Tansella M., Vazquez-Barquero J. L. (1999). Two-phase epidemiological surveys in psychiatric research. British Journal of Psychiatry.

[CR25] Duvekot J., van der Ende J., Verhulst F. C., Greaves-Lord K. (2015). The Screening accuracy of the parent and teacher-reported social responsiveness scale (SRS): Comparison with the 3Di and ADOS. Journal of Autism and Developmental Disorders.

[CR26] Dworzynski K., Ronald A., Bolton P., Happe F. (2012). How different are girls and boys above and below the diagnostic threshold for autism spectrum disorders?. Journal of the American Academy of Child and Adolescent Psychiatry.

[CR27] Epstein N. B., Baldwin L. M., Bishop D. S. (1983). The Mcmaster family assessment device. Journal of Marital and Family Therapy.

[CR28] EuroQol G. (1990). EuroQol–a new facility for the measurement of health-related quality of life. Health Policy (Amsterdam, Netherlands).

[CR29] Eyberg S. M., McDiarmid Nelson M., Duke M., Boggs S. R. (2009). Manual for the dyadic parent–child interaction coding system.

[CR30] Falkmer T., Anderson K., Falkmer M., Horlin C. (2013). Diagnostic procedures in autism spectrum disorders: A systematic literature review. European Child and Adolescent Psychiatry.

[CR31] Garnefski N., Kraaij V. (2007). The cognitive emotion regulation questionnaire-psychometric features and prospective relationships with depression and anxiety in adults. European Journal of Psychological Assessment.

[CR32] Gotham K., Risi S., Pickles A., Lord C. (2007). The autism diagnostic observation schedule: Revised algorithms for improved diagnostic validity. Journal of Autism and Developmental Disorders.

[CR33] Gulsrud A. C., Jahromi L. B., Kasari C. (2010). The co-regulation of emotions between mothers and their children with autism. Journal of Autism and Developmental Disorders.

[CR34] Hakkaart-van Roijen L., Zwirs B. W., Bouwmans C., Tan S. S., Schulpen T. W., Vlasveld L. (2007). Societal costs and quality of life of children suffering from attention deficient hyperactivity disorder (ADHD). European Child and Adolescent Psychiatry.

[CR35] Hayes S. A., Watson S. L. (2013). The impact of parenting stress: a meta-analysis of studies comparing the experience of parenting stress in parents of children with and without autism spectrum disorder. Journal of Autism and Developmental Disorders.

[CR36] Hendriksen J. G. M., Hurks P. P. M. (2009). WPPSI-III-NL Wechsler preschool and primary scale of intelligence; nederlandse bewerking.

[CR37] Herring S., Gray K., Taffe J., Tonge B., Sweeney D., Einfeld S. (2006). Behaviour and emotional problems in toddlers with pervasive developmental disorders and developmental delay: associations with parental mental health and family functioning. Journal of Intellectual Disability Research.

[CR38] Insel T., Cuthbert B., Garvey M., Heinssen R., Pine D. S., Quinn K. (2010). Research domain criteria (RDoC): Toward a new classification framework for research on mental disorders. American Journal of Psychiatry.

[CR39] International Labour Organization (2012). International standard classification of occupations (ISCO-08). Volume 1: structure, group definitions and correspondence tables.

[CR40] Jaddoe V. W., van Duijn C. M., Franco O. H., van der Heijden A. J., van Iizendoorn M. H., de Jongste J. C. (2012). The Generation R Study: Design and cohort update 2012. European Journal of Epidemiology.

[CR41] Karst J. S., Van Hecke A. V. (2012). Parent and family impact of autism spectrum disorders: A review and proposed model for intervention evaluation. Clinical Child and Family Psychology Review.

[CR42] Kim Y. S., Fombonne E., Koh Y. J., Kim S. J., Cheon K. A., Leventhal B. L. (2014). A comparison of DSM-IV pervasive developmental disorder and DSM-5 autism spectrum disorder prevalence in an epidemiologic sample. Journal of the American Academy of Child and Adolescent Psychiatry.

[CR43] Kim Y. S., Leventhal B. L., Koh Y. J., Fombonne E., Laska E., Lim E. C. (2011). Prevalence of autism spectrum disorders in a total population sample. American Journal of Psychiatry.

[CR44] Kogan M. D., Strickland B. B., Blumberg S. J., Singh G. K., Perrin J. M., van Dyck P. C. (2008). A national profile of the health care experiences and family impact of autism spectrum disorder among children in the United States, 2005–2006. Pediatrics.

[CR45] Kort W., Schittekatte M., Dekker P. H., Verhaeghe P., Compaan E. L., Bosmans M., Vermeir G. (2005). Wechsler intelligence scale for children-III. Nederlandstalige Uitgave.

[CR46] Kraaij V., Garnefski N., Schroevers M. J., van der Veek S. M. C., Witlox R., Maes S. (2008). Cognitive coping, goal self-efficacy and personal growth in HIV-infected men who have sex with men. Patient Education and Counseling.

[CR47] Lecavalier L., Leone S., Wiltz J. (2006). The impact of behaviour problems on caregiver stress in young people with autism spectrum disorders. Journal of Intellectual Disability Research.

[CR48] Leigh J. P., Du J. (2015). Brief report: Forecasting the economic burden of autism in 2015 and 2025 in the United States. Journal of Autism and Developmental Disorders.

[CR49] Lord C., Jones R. M. (2012). Annual research review: Re-thinking the classification of autism spectrum disorders. Journal of Child Psychology and Psychiatry and Allied Disciplines.

[CR50] Lord C., Rutter M., DiLavore P. C., Risi S., Gotham K., Bishop S. L. (2012). Autism diagnostic observation schedule, Second Edition (ADOS-2). Manual (Part I).

[CR51] Mandell D. S., Wiggins L. D., Carpenter L. A., Daniels J., DiGuiseppi C., Durkin M. S. (2009). Racial/ethnic disparities in the identification of children with autism spectrum disorders. American Journal of Public Health.

[CR52] Mann R. E., Sobell L. C., Sobell M. B., Pavan D. (1985). Reliability of a family tree questionnaire for assessing family history of alcohol problems. Drug and Alcohol Dependence.

[CR53] McIntosh D. N., Miller L. J., Shyu V., Dunn W. (1999). Development and validation of the short sensory profile. *Sensory Profile-NL 3 t*/*m 10 jaar. Nederlandse vertaling A. Rietman*.

[CR54] Putnam S. P., Rothbart M. K. (2006). Development of short and very short forms of the children’s behavior questionnaire. Journal of Personality Assessment.

[CR55] Santosh P. J., Mandy W. P. L., Puura K., Kaartinen M., Warrington R., Skuse D. H. (2009). The construction and validation of a short form of the developmental, diagnostic and dimensional interview. European Child and Adolescent Psychiatry.

[CR56] Simonoff E., Pickles A., Charman T., Chandler S., Loucas T., Baird G. (2008). Psychiatric disorders in children with autism spectrum disorders: prevalence, comorbidity, and associated factors in a population-derived sample. Journal of the American Academy of Child & Adolescent Psychiatry.

[CR57] Skuse D. H., Mandy W., Steer C., Miller L. L., Goodman R., Lawrence K. (2009). Social communication competence and functional adaptation in a general population of children: Preliminary evidence for sex-by-verbal IQ differential risk. Journal of the American Academy of Child and Adolescent Psychiatry.

[CR58] Slappendel G., Mandy W., van der Ende J., Verhulst F. C., van der Sijde A., Duvekot J. (2016). Utility of the 3Di short version for the diagnostic assessment of autism spectrum disorder and compatibility with DSM-5. Journal of Autism and Developmental Disorders.

[CR59] Smith I. C., Reichow B., Volkmar F. R. (2015). The Effects of DSM-5 criteria on number of individuals diagnosed with autism spectrum disorder: A systematic review. Journal of Autism and Developmental Disorders.

[CR60] Statistics Netherlands (2015). Retrieved Jul. 22, 2015 from http://www.cbs.nl/.

[CR61] Stoltenberg C., Schjolberg S., Bresnahan M., Hornig M., Hirtz D., Dahl C. (2010). The Autism birth cohort: A paradigm for gene-environment-timing research. Molecular Psychiatry.

[CR62] Sucksmith E., Roth I., Hoekstra R. A. (2011). Autistic traits below the clinical threshold: Re-examining the broader autism phenotype in the 21st century. Neuropsychology Review.

[CR63] Tellegen P. J., Winkel M., Wijnberg-Williams B. J., Laros J. A. (1998). Snijders-Oomen Niet-verbale Intelligentietest. SON-R 2½-7 Handleiding and verantwoordig [Snijders-Oomen Nonverbal Intelligence Test. SON-R 2½-7 Manual and Research Report].

[CR64] Tsai L. Y. (2012). Sensitivity and specificity: DSM-IV versus DSM-5 criteria for autism spectrum disorder. American Journal of Psychiatry.

[CR65] van Duijn G., Dijkxhoorn Y., Noens I., Scholte E., van Berckelaer-Onnes I. (2009). Vineland Screener 0–12 years research version (NL). Constructing a screening instrument to assess adaptive behaviour. International Journal of Methods in Psychiatric Research.

[CR66] Van Leeuwen K., Noens I. (2013). Parental behavior scale for autism spectrum disorders..

[CR67] van Roijen L., Essink-Bot M. L., Koopmanschap M. A., Bonsel G., Rutten F. F. (1996). Labor and health status in economic evaluation of health care. The health and labor questionnaire. International Journal of Technology Assessment in Health Care.

[CR68] Veerman J. W., Janssen J., Kroes G., De Meyer R., Nguyen L., Vermulst A. (2012). Vragenlijst Gezinsfunctioneren volgens Ouders (VGFO). Handleiding [Family Functioning Questionnaire reported by parents. Manual].

[CR69] Vermulst A., Kroes G., De Meyer R., Nguyen L., Veerman J. W. (2012). Opvoedingsbelastingvragenlijst (OBVL). Handleiding. [Parenting stress questionnaire. Manual].

[CR70] Volkmar F. R., McPartland J. C. (2014). From kanner to DSM-5: Autism as an evolving diagnostic concept. Annual Review of Clinical Psychology.

[CR71] Zaidman-Zait A., Mirenda P., Duku E., Vaillancourt T., Smith I. M., Szatmari P. (2016). Impact of personal and social resources on parenting stress in mothers of children with autism spectrum disorder. Autism: The International Journal of Research and Practice.

